# Case report: An N-of-1 study using amplitude modulated transcranial alternating current stimulation between Broca's area and the right homotopic area to improve post-stroke aphasia with increased inter-regional synchrony

**DOI:** 10.3389/fnhum.2024.1297683

**Published:** 2024-02-22

**Authors:** Erika Omae, Atsushi Shima, Kazuki Tanaka, Masako Yamada, Yedi Cao, Tomoyuki Nakamura, Hajime Hoshiai, Yumi Chiba, Hiroshi Irisawa, Takashi Mizushima, Tatsuya Mima, Satoko Koganemaru

**Affiliations:** ^1^Department of Regenerative Systems Neuroscience, Human Brain Research Center, Graduate School of Medicine, Kyoto University, Kyoto, Japan; ^2^Department of Neurobiology and Physiology, Graduate School of Medicine, Kyoto University, Kyoto, Japan; ^3^Department of Rehabilitation Medicine, Dokkyo Medical University, Tochigi, Japan; ^4^The Graduate School of Core Ethics and Frontier Sciences, Ritsumeikan University, Kyoto, Japan; ^5^Department of Rehabilitation Medicine, Hokkaido University Hospital, Sapporo, Japan

**Keywords:** transcranial alternating current stimulation, amplitude modulation, aphasia, stroke, coherence

## Abstract

Over one-third of stroke survivors develop aphasia, and language dysfunction persists for the remainder of their lives. Brain language network changes in patients with aphasia. Recently, it has been reported that phase synchrony within a low beta-band (14–19 Hz) frequency between Broca's area and the homotopic region of the right hemisphere is positively correlated with language function in patients with subacute post-stroke aphasia, suggesting that synchrony is important for language recovery. Here, we employed amplitude-modulated transcranial alternating current stimulation (AM-tACS) to enhance synchrony within the low beta band frequency between Broca's area and the right homotopic area, and to improve language function in a case of chronic post-stroke aphasia. According to an N-of-1 study design, the patient underwent short-term intervention with a one-time intervention of 15 Hz-AM-tACS with Broca's and the right homotopic areas (real condition), sham stimulation (sham condition), and 15 Hz-AM-tACS with Broca's and the left parietal areas (control condition) and long-term intervention with sham and real conditions (10 sessions in total, each). In the short-term intervention, the reaction time and accuracy rate of the naming task improved after real condition, not after sham and control conditions. The synchrony between the stimulated areas evaluated by coherence largely increased after the real condition. In the long-term intervention, naming ability, verbal fluency and overall language function improved, with the increase in the synchrony, and those improvements were sustained for more than a month after real condition. This suggests that AM-tACS on Broca's area and the right homotopic areas may be a promising therapeutic approach for patients with poststroke aphasia.

## 1 Introduction

More than one-third of stroke survivors experience aphasia with a poor prognosis. Furthermore, 30%−43% of them show persistent severe symptoms for more than 1 year after stroke onset (Bakheit et al., 2007). Their impairments include comprehension and expression of speech, reading, and writing (Brady et al., 2016), which can decrease social activities and cause them to become isolated and depressed, reducing their quality of life (Doogan et al., 2018). Speech and language therapy (SLT) has been widely implemented and recommended for treating aphasia for over half a century (Chapey, 2008). However, SLT shows moderate effects, even when administered at high intensity, depending on individual symptoms (Brady et al., 2016; Breitenstein et al., 2017). A novel strategy has been sought to achieve a greater therapeutic effect in aphasia.

Previous neuroimaging studies have shown that the right hemisphere facilitates language recovery, possibly by releasing interhemispheric inhibition from the damaged left hemisphere during recovery from post-stroke aphasia (Hamilton et al., 2011). Recently, it was reported that phase synchrony within the low beta-band (14–19 Hz) frequency between Broca's area and the homotopic region of the right hemisphere is positively correlated with language function in subacute post-stroke aphasia (Kawano et al., 2021). We hypothesized that enhancing synchrony within the low beta-band frequency between Broca's area and the homotopic region of the right hemisphere could improve the language neural network and language function in post-stroke aphasia. Transcranial alternating current stimulation (tACS) is a noninvasive brain stimulation (NIBS) method that uses sinusoidal alternating electric currents to affect cortical oscillatory neuronal activity. tACS synchronizes brain oscillations, induces long-term synaptic plasticity, and promotes functional recovery in patients with neurological diseases (Fröhlich and McCormick, 2010; Ozen et al., 2010; Reato et al., 2010; Ali et al., 2013; Koganemaru et al., 2018, 2019, 2020; Negahbani et al., 2018; Nojima et al., 2023; Shima et al., 2023a,b).

Recently, amplitude-modulated tACS (AM-tACS) was developed (Witkowski et al., 2016; Negahbani et al., 2018). It has two components of stimulation waveforms: high-frequency (>100 Hz) sinusoidal carrier frequency and low-frequency (e.g., 10–15 Hz) amplitude modulation as the envelope. Low-frequency amplitude modulation has been reported to modulate neuronal oscillations as well as low-frequency tACS (Chander et al., 2016; Witkowski et al., 2016; Minami and Amano, 2017; Esmaeilpour et al., 2021). Furthermore, the amplitude modulation phase almost coincides between the two stimulated regions when using the two electrodes, suggesting that it would help enhance inter-regional brain synchrony. While the use of beta band tACS has been limited due to phosphene induction, AM-tACS does not induce phosphenes, according to previous reports (Chander et al., 2016; Minami and Amano, 2017). Therefore, we used AM-tACS to enhance the synchrony between Broca's area and the homotopic region of the right hemisphere. We systematically compared the short- and long-term effects in a patient with chronic post-stroke aphasia according to an N-of-1 study design.

## 2 Case description

### 2.1 Patient characteristics

A 76-year-old man with nonfluent aphasia due to left cerebral infarction was referred to Kyoto University Hospital. At the age of 69 years, he was admitted to the hospital with acute onset of nonfluent aphasia with right mild hemiparesis. Head magnetic resonance imaging (MRI) revealed left cerebral infarction in the territory of left middle cerebral artery, including the left operculum (Table 1). His medical history included myocardial infarction and diabetes mellitus.

**Table 1:** Clinical course of the case.

**Months**	**Clinical findings**
0	Admission to the hospital for acute onset of difficulty in walking followed by aphasia and right hemiparesis • Magnetic resonance imaging (MRI) revealed acute cerebral infarction at the territory of the left middle cerebral artery
1.5	Transferred to the rehabilitation hospital
3	Discharged from the hospital • Aphasia remained • Right hemiparesis recovered
87	Participated in the study

### 2.2 Therapeutic intervention

**Figure 1:**
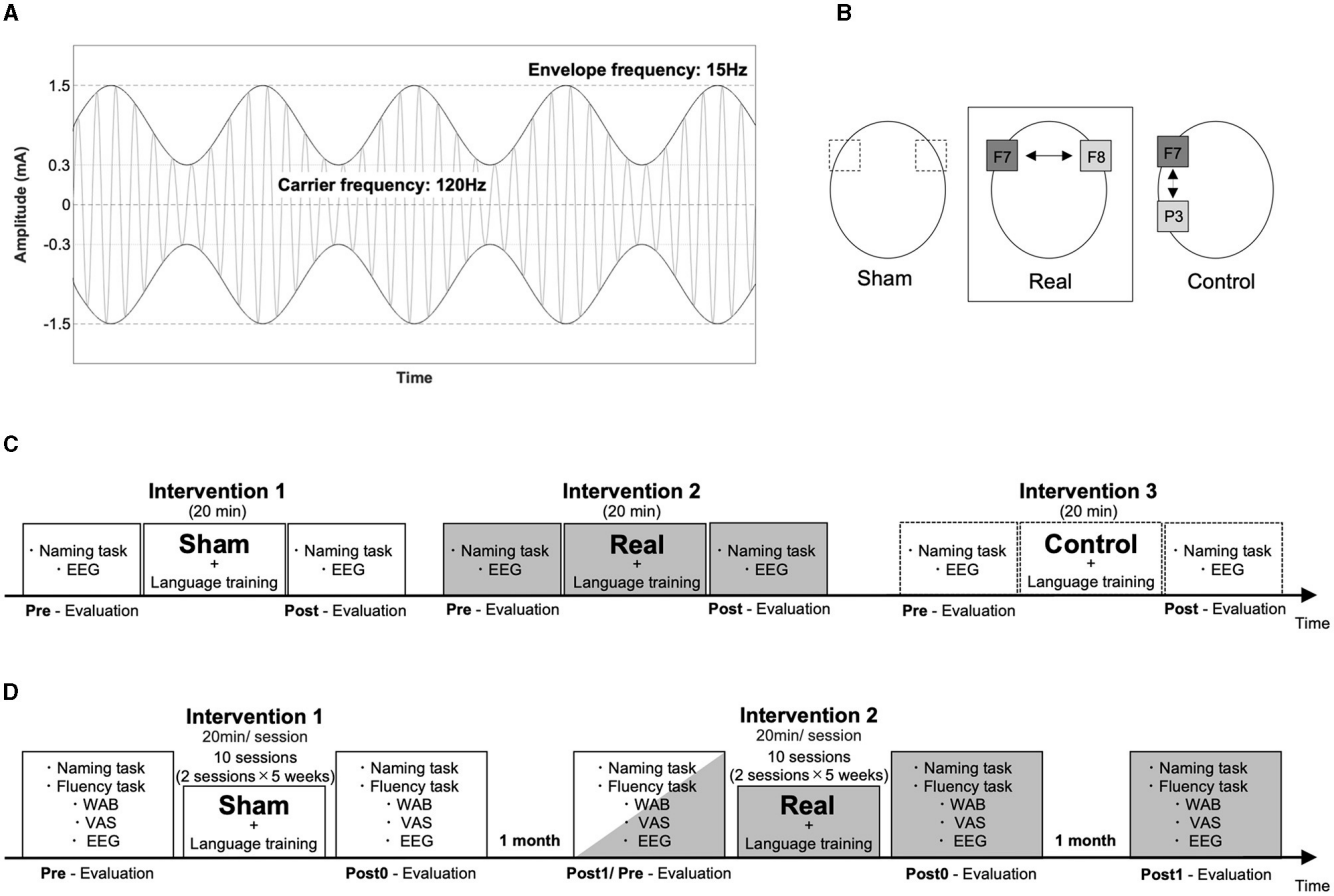
Experimental setup. The stimulator output signal during AM-tACS is illustrated with amplitude of a carrier signal (120 Hz) modulated at 15 Hz of a signal envelop **(A)**. The electrodes (5 × 5 cm) were placed on F7 and F8 in the sham and real conditions, and on F7 and P5 in the control condition **(B)**. The order of the interventions is illustrated in the short-term and long-term interventions **(C, D)**.

We conducted three types of interventions to examine the effect of AM-tACS according to an N-of-1 study design: (1) AM-tACS with 120 Hz frequency of sinusoidal carrier waves with 15 Hz of sinusoidal amplitude modulation, a peak-to-peak amplitude of 3 mA (−1.5–1.5 mA) and trough amplitudes of ± 0.3 mA (Figure 1A) on the Broca's area [centering the 5 × 5 cm^2^ electrode on F7 according to the international 10–20 electroencephalography (EEG) system] and on the homotopic region of the right hemisphere (centering the 5 × 5 cm^2^ electrode on F8; real condition), (2) sham AM-tACS with only 10 cycles of 15 Hz amplitude modulation of AM-tACS given on the same regions with the real condition (sham condition), and (3) AM-tACS with the Broca's area and the left parietal area using the same stimulation parameter with the real condition (control condition) by using Nurostym tES (Neuro Device Group S.A., Warsaw, Poland; Figure 1B). The stimulation sites were confirmed using a neuronavigation system with the patient's head MRI (Brainsight Brainbox Ltd., Cardiff, UK). For the short-term intervention, one-time sessions of sham, real, and control conditions were performed. Each stimulation was applied for 20 min and combined with a 20-min language training session including a naming task, different from that used in the evaluation. The interval between sessions was over 1 h, and the control condition was performed 1 day after the real and sham conditions because of patient fatigue (Figure 1C). For the long-term intervention, we performed 10 sessions of sham and real conditions (one session per day, 2 days per week for 5 weeks) with the interval of 1 month (Figure 1D).

## 3 Assessments

### 3.1 Clinical measurements

During the short-term intervention, clinical evaluations were performed before and immediately after one session of each condition (pre- and post-intervention, respectively). During the long-term intervention, they were performed before, within 1 week after, and 1 month after the total 10 sessions of each condition (pre, post0, and post1, respectively). The assessment of language function included the reaction time and accuracy rate of a naming task in a short-term intervention. The patient was asked to name as immediately the 12 line drawings extracted from the Snodgrass and Nishimoto pictures (Snodgrass and Vanderwart, 1980; Nishimoto et al., 2005) shown on a 27-inch monitor in front of him as possible. The sets of line drawings were different in each condition to prevent learning effects, while they were selected with almost the same degree of familiarity and number of morae. In the long-term intervention, we evaluated the reaction time and accuracy rate of the naming task, verbal fluency, aphasia quotient of the Western Aphasia Battery (WAB) (Kertesz, 1982). The aphasia quotient is a composite score indicated by the percentile, which provides an overall measure of aphasia severity, in which lower scores denote more severe aphasia (the maximal score is 100) and visual analog scale (VAS) for the patient's subjective assessment of the general language function (score was determined by the distance on the 10 cm line; “0” indicated the worst condition and “10” indicated the best condition). The naming task comprised 24 line drawings extracted from Snodgrass and Nishimoto's pictures. The reaction time was measured by the time interval from the onset time of the drawing on the monitor to the time for him to name it by using the video-recording (60 fps). In the verbal fluency task, the patient was asked to name as many items that begin with a certain letter of the Japanese syllabary characters, “Hiragana” [the sound of “Ka (k∧)” and “A (∧)” in the sham and real conditions, respectively] and as various items of a given semantic category (“vegetables” and “vehicles” in the sham and real conditions, respectively) as possible. The number of named items was scored for each character and category. All the clinical evaluations were double-blinded.

### 3.2 Electroencephalography recording

We recorded EEG signals and measured the coherence within the stimulated areas to evaluate interhemispheric synchrony in the language network. The patients were seated comfortably in an armchair. The EEG signals were recorded using 64 electrodes by eegoTM sports (ANT Neuro, Hengelo, Netherlands) during 3-min resting with eyes open. EEG signals were amplified using an Eego sports amplifier. Electrodes M1 and M2 were selected as references (Kawano et al., 2021). The impedance of all the electrodes was < 15 kΩ. The data were recorded and saved at a sampling rate of 2 kHz.

### 3.3 Data analysis

#### 3.3.1 Preprocessing

We removed artifacts of the blink, electrooculographic activities, and muscle activities from the EEG signals using independent component analysis (ICA) (Hyvärinen and Oja, 2000) with the EEGLAB MATLAB toolbox including the artifact subspace reconstruction method, which detects the time windows of signals with significant artifacts (Pedroni et al., 2019), and “ICLabel,” which automatically distinguishes independent components (ICs) as brain or non-brain sources according to a large number of crowd-sourced IC labels (Pion-Tonachini et al., 2019).

#### 3.3.2 Coherence between Broca's area and the right homotopic area

We then calculated the power spectral density of the EEG using a fast Fourier transform (FFT). We applied the FFT to 1,000 ms segments with a 50 ms time shift. The evaluated frequency range was 5–40 Hz. For the coherence analysis, using FFT, we computed the cross- and auto-spectra in the frequency domain of the EEG signals within a frequency range of 14–16 Hz in Broca's area (F7) and the right homotopic area, including AF8, F6, FC6, F8, and FT8, which were stimulated by AM-tACS. Coherence is defined as cross-spectra normalized by auto-spectra. It is expressed by the following equation (1), where *f*_xx_(*j*), *f*_yy_(*j*), and |*f*_xy_(*j*)| denote the auto-spectra and cross-spectra at frequency *j* (Mima et al., 2001).


(1) $$\begin{array}{l} {{|R}_{xy}\left(j\right)|}^{2}=\frac{{\left|f_{xy}\left(j\;\right)\right|}^{2}}{f_{xx}\left(j\right)f_{yy}\left(j\right)} \end{array}$$

To stabilize the variance, we applied an arc hyperbolic tangent transformation to the coherence according to the following equation (2):


(2) $$\begin{array}{l} \tanh^{-1}|R_{xy}\left(j\right)|=\frac{1}{2}\ln\left(\frac{1+|R_{xy}\left(j\right)|}{1-|R_{xy}\left(j\right)|}\;\right) \end{array}$$

The average of the arc hyperbolic tangents transforming the coherences of F7 with AF8, F6, FC6, F8, and FT8 was calculated as an index of synchrony.

## 4 Results

No adverse or unanticipated events developed during the short- or long-term interventions. The patient did not experience sensations like phosphenes, cutaneous irritation, or pain.

### 4.1 Short-term intervention

**Figure 2:**
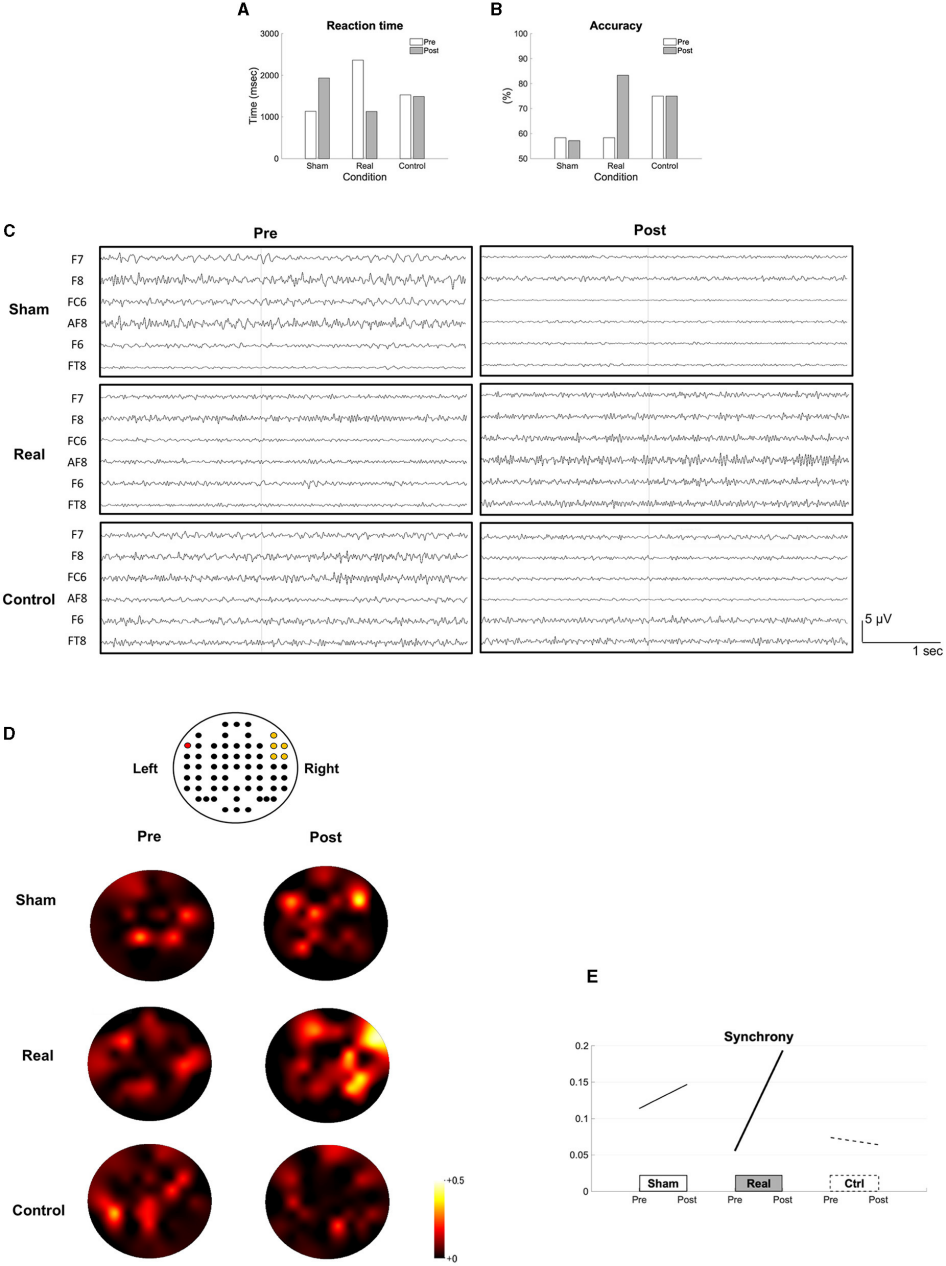
Results of the short-term intervention. The reaction time **(A)**, the accuracy rate **(B)** in the naming task, the recorded EEG data **(C)**, the topomaps of the arc hyperboric tangent of the coherence **(D)** and the synchrony **(E)** are shown before (pre) and after each intervention (post). The EEG channel location (62 channels excluding M1 and M2) is illustrated above the topomaps. The red colored channel is F7 and the yellow colored channels are AF8, F6, F8, FC6, and FT8 from top to bottom, and left to right **(D)**. The synchrony between the Broca's and the right homotopic areas is indicated by the average of the arc hyperbolic tangents transformation applied to the coherences between F7 and AF8, F7-F6, F7-FC6, F7-F8, and F7-FT8 **(E)**.

For the naming task, the reaction time was shortened (Figure 2A), and the accuracy rate improved after the real condition (Figure 2B). The recorded EEG signals in each condition were shown in the Figure 2C. The synchrony between Broca's area and the right homotopic area largely increased after the real but not after the sham and control conditions (Figures 2D, E).

### 4.2 Long-term intervention

**Figure 3:**
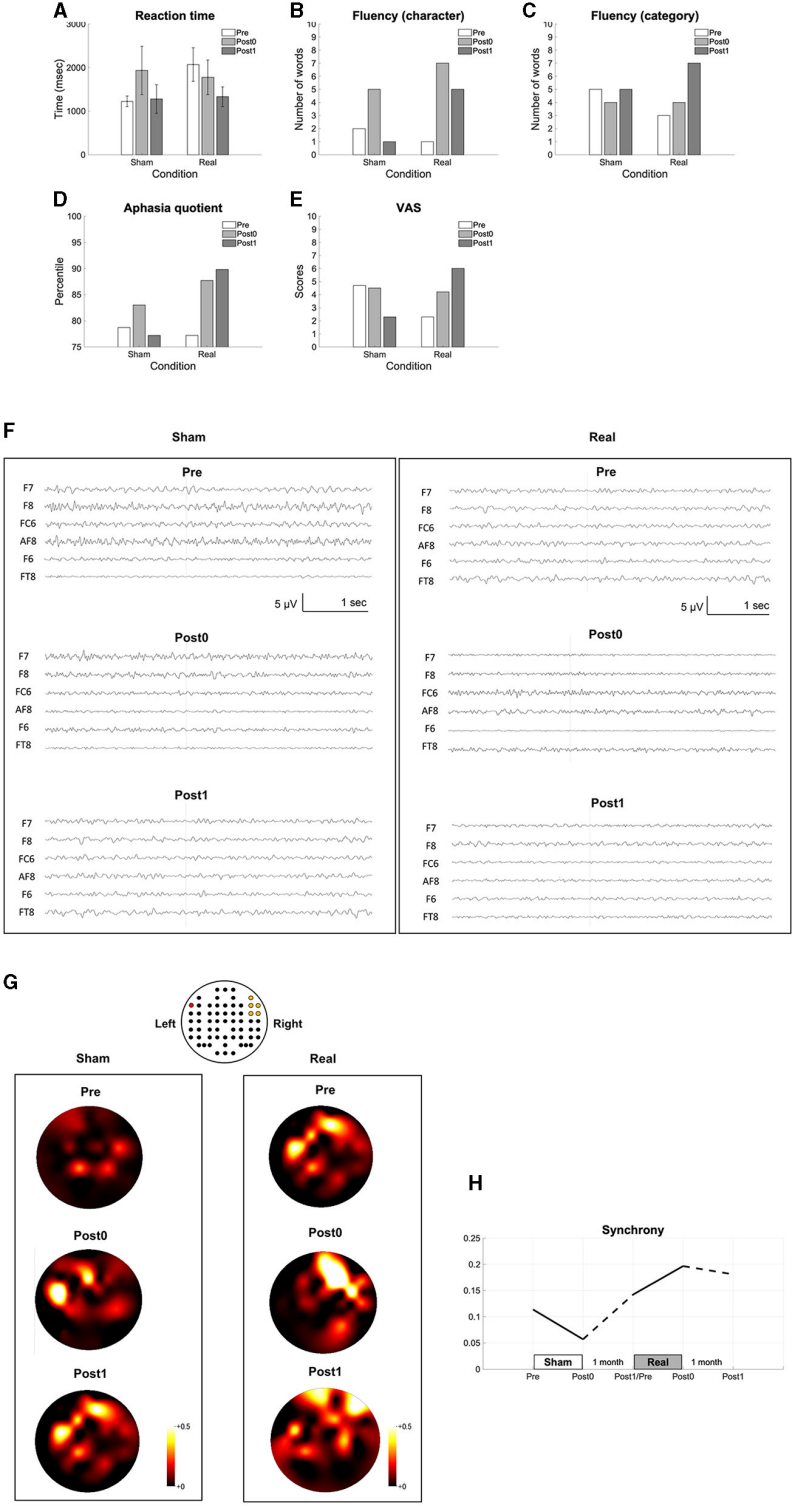
Results of the long-term intervention. The reaction time in the naming task **(A)**, the number of words in the verbal fluency task **(B, C)**, the aphasia quotient **(D)**, VAS **(E)**, the recorded EEG data **(F)**, the topomaps of the arc hyperboric tangent of the coherence **(G)** and the synchrony between the Broca's and the right homotopic areas indicated by the averaged arc hyperbolic tangents transformation applied to the coherences between F7 and AF8, F7-F6, F7-FC6, F7–F8, and F7–FT8 **(H)** are shown before (pre), within a week after (post0), and 1 month after the intervention (post1). The EEG channel location is illustrated above the topomaps in the same way as in Figure 2 **(G)**.

We found improvements in language functions evaluated using naming (Figure 3A), verbal fluency tasks (Figures 3B, C), and the aphasia quotient of the WAB (Figure 3D) after the real condition, but not after the sham condition. The VAS scores also improved after real condition (Figure 3E). The recorded EEG signals in each condition were shown in the Figure 3F. All clinical improvements were sustained for a month after the real condition. In addition, we found that synchrony increased and sustained for a month after the real condition (Figures 3G, H).

## 5 Discussion

The present case demonstrates the potential therapeutic effect of 15 Hz AM-tACS on Broca's area and the homotopic region of the right hemisphere combined with SLT. The short-term evaluation showed that it was effective in improving naming ability along with enhancing synchrony between the stimulated areas. In contrast, sham- or AM-tACS on Broca's area and the left parietal area did not improve them. The long-term intervention showed sustained improvements in general language function evaluated using WAB, naming ability, verbal fluency, and subjective assessment of language function, along with increased synchrony between Broca's area and the homotopic region of the right hemisphere.

Although right hemisphere recruitment may be insufficient for overall language recovery, it may facilitate recovery by releasing interhemispheric inhibition from the damaged left hemisphere (Hamilton et al., 2011). As for oscillatory brain activity, the overall connectivity of Broca's area at beta oscillation frequencies correlates with future clinical improvement in patients with aphasia (Nicolo et al., 2015). During a sentence completion task, increased power in the right hemisphere was observed within a low beta-band frequency compared with that in the left hemisphere in patients with chronic post-stroke aphasia (Lima et al., 2023). Synchrony within a low beta-band frequency between Broca's area and the homotopic region of the right hemisphere was decreased compared with that in healthy controls. It was positively correlated with language function in patients with subacute post-stroke aphasia (Kawano et al., 2021). These studies suggest that language function migrates to the non-language-dominant hemisphere in the recovery of language function and that the functional migration associated with language ability may be indicated, especially by low-beta interhemispheric oscillatory synchronization. Therefore, 15 Hz AM-tACS enabling phase-synchronized stimulation on the two given regions enhanced the synchrony within a low beta-band frequency between Broca's area and the homotopic region of the right hemisphere, suggesting that increased interhemispheric connectivity led to improved language function in this case. Further, 15 Hz AM-tACS on Broca's area and the left parietal area did not induce these effects, suggesting that enhancement of synchrony between Broca's area and the right homotopic region is important for language recovery.

Interhemispheric homotopic functional connectivity significantly decreases after stroke, and this decrease is strongly associated with behavioral impairment in post-stroke patients, including aphasia (Siegel et al., 2016). Furthermore, the longitudinal normalization of decreased interhemispheric functional connectivity is associated with clinical recovery (Carrera and Tononi, 2014). Therefore, enhanced interhemispheric synchrony associated with specific functions may be a potential therapeutic target for AM-tACS.

Our findings suggest that the combination of AM-tACS and SLT is appropriate. Combined with repetitive rehabilitation programs, NIBS can enhance functional recovery by inducing associative plasticity (Koganemaru et al., 2015). Although some studies on tDCS combined with SLT have shown language recovery in aphasia (Kang et al., 2011; You et al., 2011), the effects have been inconsistent. Thus, NIBS that targets specific functional networks would be more appropriate for inducing therapeutic effects.

In this case, the long-term intervention of repetitive sessions of SLT alone (sham condition) induced a partial improvement in language function within a week after the end of the intervention along with decreased inter-hemispheric synchrony. Further, these clinical improvements were not sustained for more than a month. This finding coincides with a previous report showing a limited effect of intensive SLT on chronic post-stroke aphasia (Breitenstein et al., 2017). In contrast, combining AM-tACS with SLT induced sustained improvement in language function for more than a month after the end of the intervention with increased inter-hemispheric synchrony. Thus, combining AM-tACS with SLT may induce long-lasting associative plasticity in the functional network required for sustained language recovery.

This case report provides a novel finding that a low-beta band frequency AM-tACS on Broca's area and the right homotopic area to enhance interhemispheric synchrony may be a promising rehabilitative approach to induce long-lasting improvement in language function in post-stroke aphasia. A further study with a larger number of patients would be necessary.

## 6 Patient perspective

The patient stated that he felt the improvement in fluency of words during daily conversation with others after the long-term intervention with real condition and hoped that the present findings may contribute to the development of new strategies for the treatment of aphasia.

## Data availability statement

The raw data supporting the conclusions of this article will be made available by the authors, without undue reservation.

## Ethics statement

The studies involving humans were approved by the Hokkaido University Certified Review Board. The studies were conducted in accordance with the local legislation and institutional requirements. The patient provided his written informed consent to participate in this study. Written informed consent was obtained from the patient for the publication of any data included in this article.

## Author contributions

EO: Data curation, Investigation, Visualization, Writing—original draft. AS: Data curation, Investigation, Writing—review & editing. KT: Investigation, Writing—review & editing. MY: Data curation, Investigation, Writing—review & editing. YCa: Data curation, Investigation, Writing—review & editing. TN: Data curation, Investigation, Writing—review & editing. HH: Data curation, Investigation, Writing—review & editing. YCh: Data curation, Investigation, Writing—review & editing. HI: Investigation, Supervision, Writing—review & editing. TMiz: Project administration, Supervision, Writing—review & editing. TMim: Funding acquisition, Methodology, Validation, Writing—review & editing. SK: Conceptualization, Data curation, Formal analysis, Funding acquisition, Investigation, Methodology, Project administration, Validation, Writing—review & editing.
