# Supplementary material for: Case report: An N-of-1 study using amplitude modulated transcranial alternating current stimulation between Broca's area and the right homotopic area to improve post-stroke aphasia with increased inter-regional synchrony
Source: Front Hum Neurosci. 2024 Feb 22;18:1297683. doi: 10.3389/fnhum.2024.1297683 (PMC10917932; doi:10.3389/fnhum.2024.1297683)
Supplement: Supplementary file 1 [file Data_Sheet_1.pdf]

CARE case report guidelines

CARE Checklist of information to include when writing a case report

(cc) BY-NC-ND

Topic	Item	Checklist item description	Reported on Line
Title	1	The diagnosis or intervention of primary focus followed by the words "case report"	✔
Key Words	2	2 to 5 key words that identify diagnoses or interventions in this case report, including "case report"	✔
Abstract (no references)	3a	Introduction: What is unique about this case and what does it add to the scientific literature?	✔
	3b	Main symptoms and/or important clinical findings	✔
	3c	The main diagnoses, therapeutic interventions, and outcomes	✔
	3d	Conclusion—What is the main "take-away" lesson(s) from this case?	✔
Introduction	4	One or two paragraphs summarizing why this case is unique (**may include references**)	✔
Patient Information	5a	De-identified patient specific information.	✔
	5b	Primary concerns and symptoms of the patient.	✔
	5c	Medical, family, and psycho-social history including relevant genetic information	✓
	5d	Relevant past interventions with outcomes	NA
Clinical Findings	6	Describe significant physical examination (PE) and important clinical findings.	✔
Timeline	7	Historical and current information from this episode of care organized as a timeline	✔
Diagnostic Assessment	8a	Diagnostic testing (such as PE, laboratory testing, imaging, surveys).	✔
	8b	Diagnostic challenges (such as access to testing, financial, or cultural)	NA
	8c	Diagnosis (including other diagnoses considered)	✔
	8d	Prognosis (such as staging in oncology) where applicable	NA
Therapeutic Intervention	9a	Types of therapeutic intervention (such as pharmacologic, surgical, preventive, self-care)	✓
	9b	Administration of therapeutic intervention (such as dosage, strength, duration)	✔
	9c	Changes in therapeutic intervention (with rationale)	✔
Follow-up and Outcomes	10a	Clinician and patient-assessed outcomes (if available)	✔
	10b	Important follow-up diagnostic and other test results	✔
	10c	Intervention adherence and tolerability (How was this assessed?)	✔
	10d	Adverse and unanticipated events	✔
Discussion	11a	A scientific discussion of the strengths AND limitations associated with this case report	✔
	11b	Discussion of the relevant medical literature **with references**.	✔
	11c	The scientific rationale for any conclusions (including assessment of possible causes)	✔
	11d	The primary "take-away" lessons of this case report (without references) in a one paragraph conclusion	✔
Patient Perspective	12	The patient should share their perspective in one to two paragraphs on the treatment(s) they received	✔
Informed Consent	13	Did the patient give informed consent? Please provide if requested	Yes ☑ No ☐
